# Supplementary material for: Chemogenetic ON and OFF switches for RNA virus replication
Source: Nat Commun. 2021 Mar 1;12:1362. doi: 10.1038/s41467-021-21630-5 (PMC7921684; doi:10.1038/s41467-021-21630-5)
Supplement: Supplementary file 2 — Supplementary Information [file 41467_2021_21630_MOESM2_ESM.pdf]

Supplementary Materials

Chemogenetic ON and OFF switches for RNA virus replication

Heilmann et al.

- Supplementary Figure 1
- Supplementary Figure 2
- Supplementary Figure 3
- Supplementary Figure 4
- Supplementary Figure 5
- Supplementary Figure 6
- Supplementary Figure 7
- Supplementary Figure 8
- Supplementary Figure 9
-
- Supplementary Table 1

Corresponding authors:

dorothee.von-Laer@i-med.ac.at
guido.wollmann@i-med.ac.at

Supplementary Figure 1

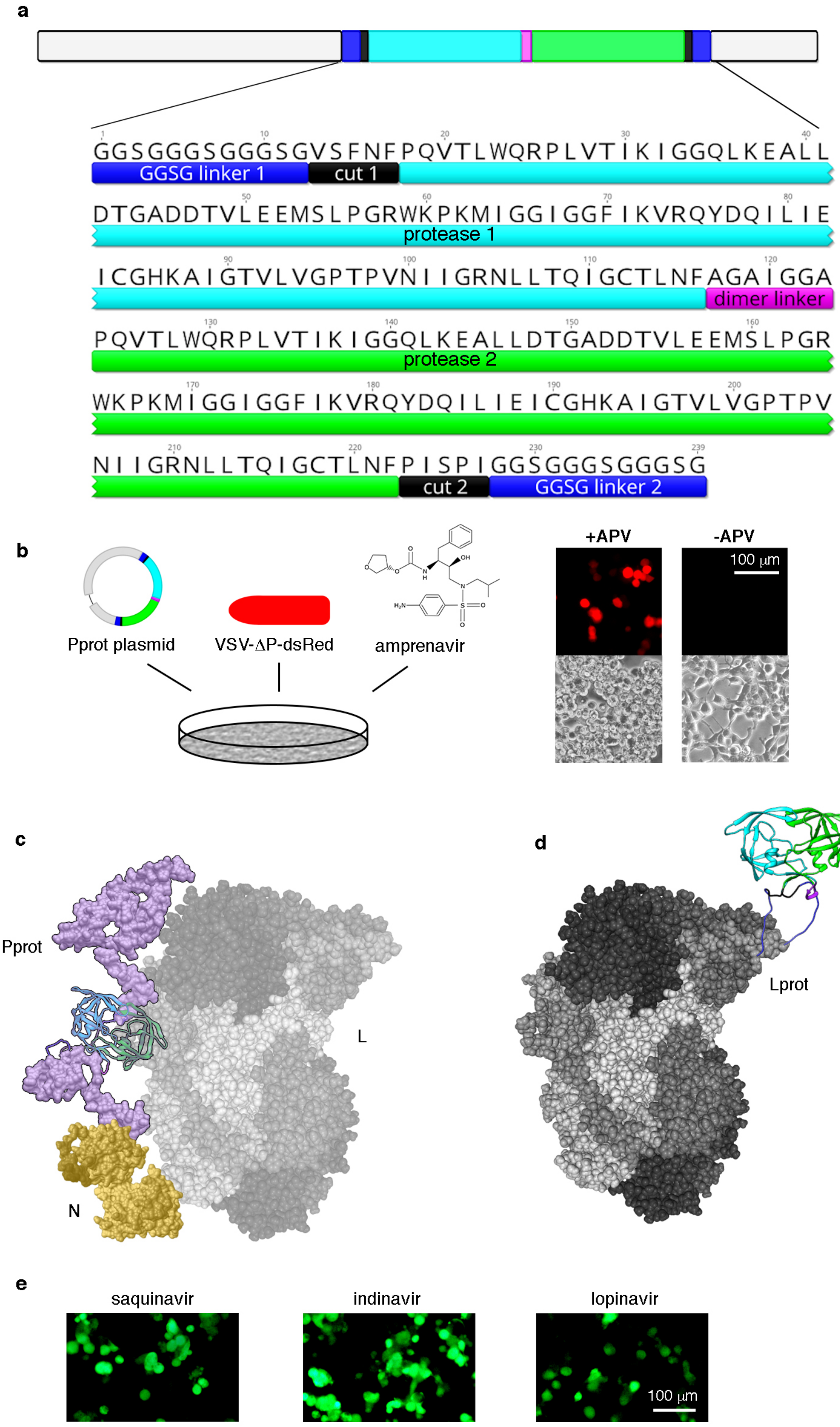

Supplementary Fig. 1. Prot-ON constructs and effect of protease inhibitors. a) Amino acid sequence of the protease construct insert containing a linked (purple) dimer HIV protease (green and turquois) flanked by autoproteolytic cleavage sites

(black) and GGSG linkers (blue). **b)** A mini-genome assay on 293T cells consisting of a plasmid-encoded Pprot construct and a phosphoprotein-deleted VSV-ΔP-dsRed was applied as proof-of-concept for conditional viral gene expression depending on presence of protease inhibitor amprenavir. Red fluorescence reporter signal indicates effective gene expression only in cells treated with APV. Mini-genome assay was performed two times independently with two wells per condition. **c)** Model depiction of the NPL replication complex in the presence of PI. **d)** Structure prediction of Lprot with the protease dimer construct inserted into the methyltransferease domain at position aa1620. **e)** BHK-21 cells were infected with VSV-Pprot-GFP at MOI 1 in the presence of various HIV protease inhibitors (saquinavir, indinavir, lopinavir). Representative photomicrographs were taken 24 hours post infection. Each of the protease inhibitors was tested in at least two independent experiments.

Supplementary Figure 2

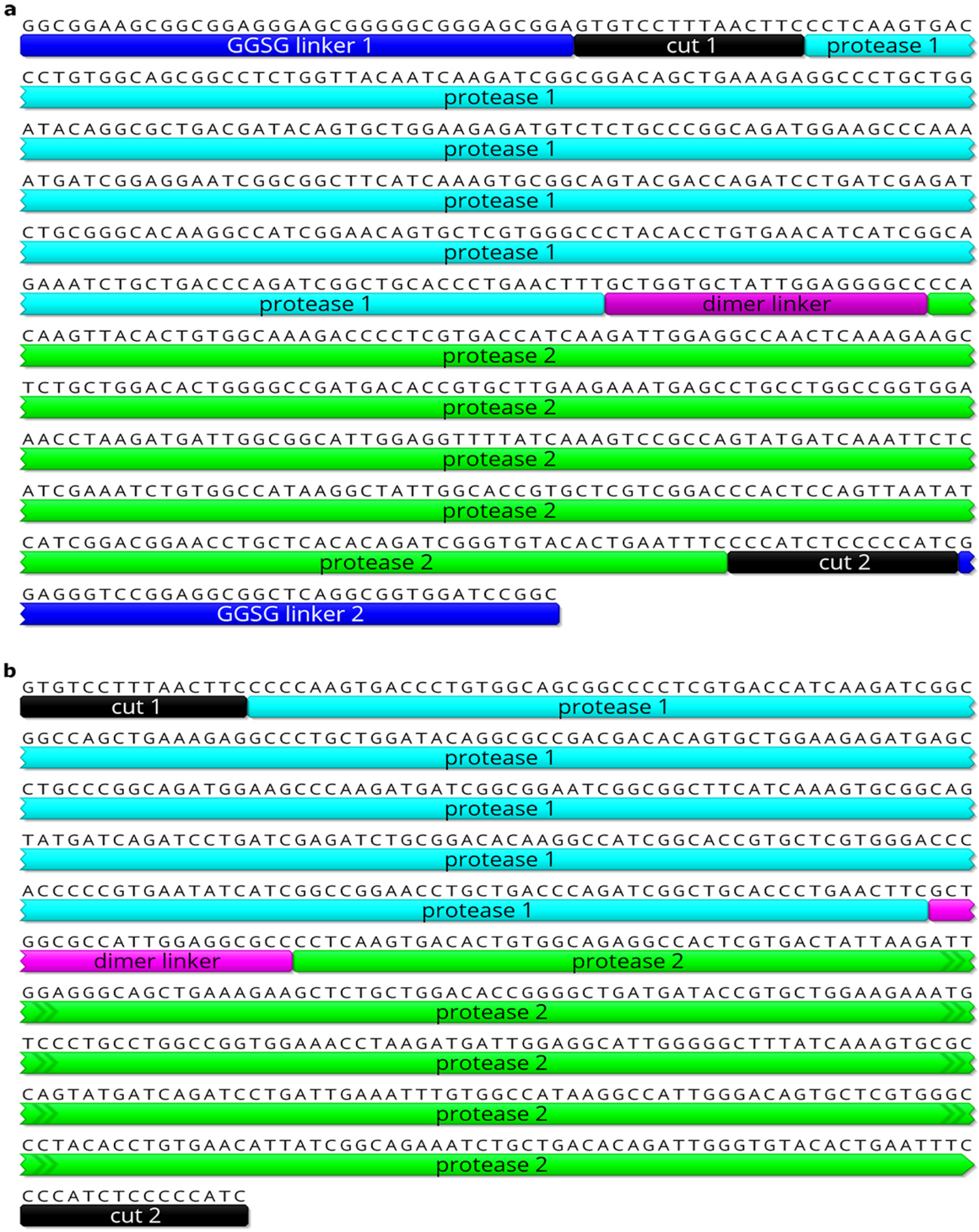

Supplementary Fig. 2. Sequence variants of HIV protease linked dimer construct. a) Codon-usage and homology-optimized HIV protease linked dimer. Genbank accession number: MW316665. **b)** Non-optimized HIV protease linked dimer. Genbank accession number: MW316666. Purple: dimer linker; blue: GGSG linkers: 3x GGSG; black: HIV protease cleavage sequence; green / turquois: proteases.

Supplementary Figure 3

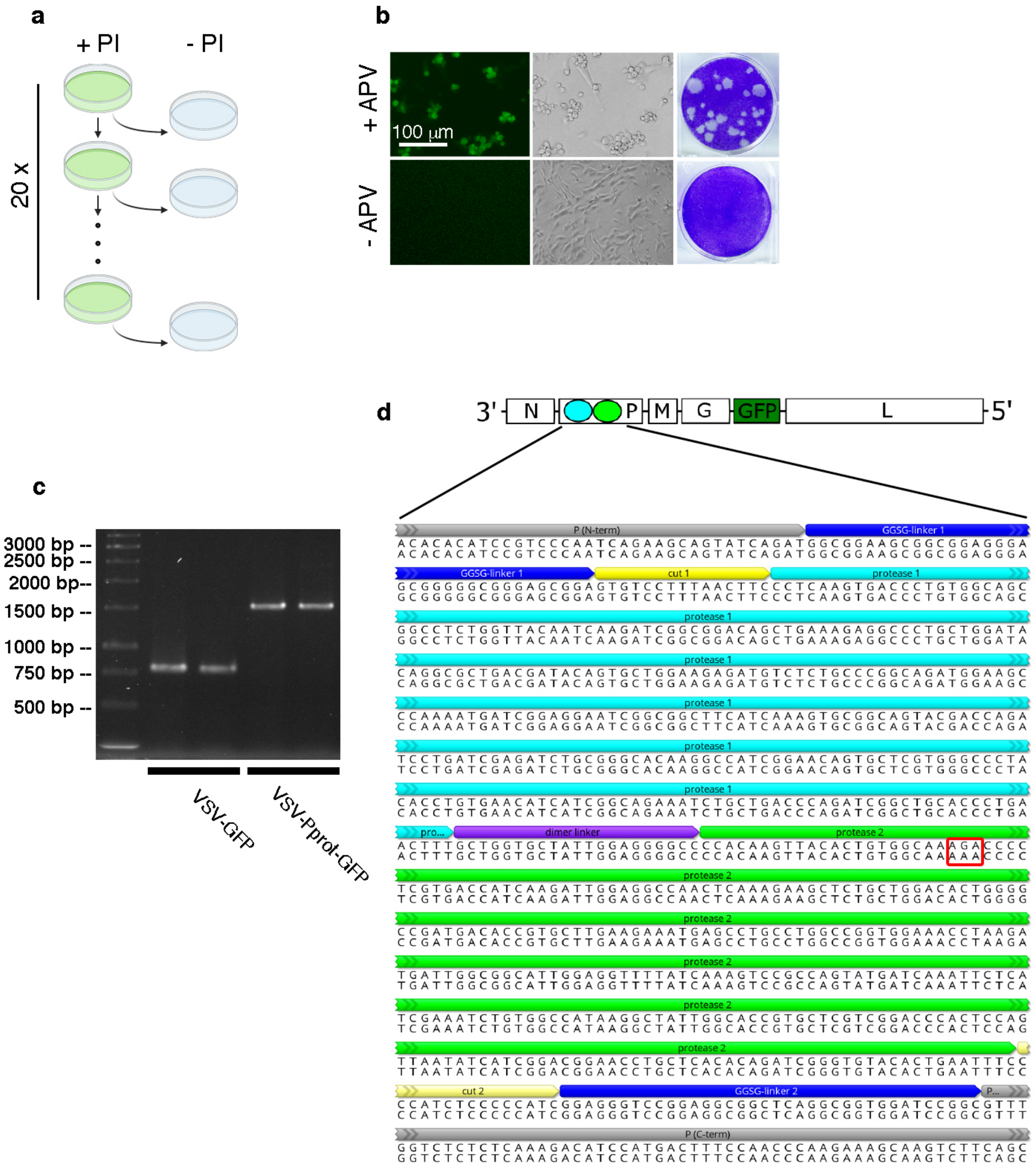

Supplementary Fig. 3. Genetic stability of protease inhibitor dependency of VSV-Pprot. VSV-Pprot-GFP was serially passaged 20 times on BHK-21 cells in the presence of either optimal (10 µM) or suboptimal (1 µM) doses of APV. In parallel, 5 µl of each supernatant was transferred onto dishes without APV. Experiment was performed with two independent passage lines **(a)**. After 20 passages GFP expression and plaque forming capacity remained dependent on presence of APV **(b).** Viral genomic RNA collected from passage P20 from the suboptimal amprenavir-treated virus propagation was purified, reverse transcribed and a PCR (primer pair P-for / M-rev) performed on the insert P-196PR2 (size: 1491 bp). A VSV variant without protease insertion was used as negative control (size: 774 bp). Pairwise bands represent two independent passage studies **(c).** Sequencing of the insert after 20 passages revealed a single mutation (red frame) with no effect on the ability to control virus activity **(d)**. Schematic in **(a)** generated with BioRender. Source data are provided in the Source Data File.

Supplementary Figure 4

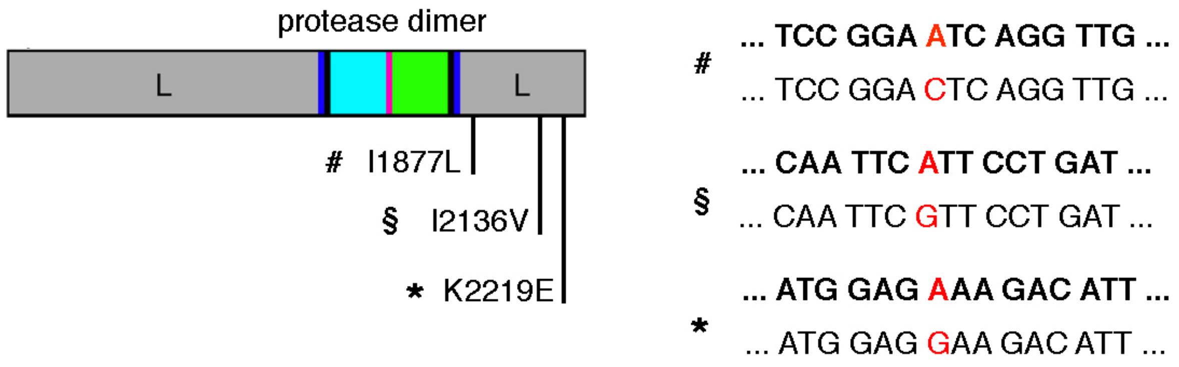

Supplementary Fig. 4. Secondary mutations in VSV-Lprot. Non-synonymous mutations in L-protein of VSV-Lprot-GFP. Amino acid changes respective to amino acid numbering from L start codon with identifiers (#, §, *) to nucleotide sequences. Reference nucleotide sequence in bold. Mutations are highlighted in red.

Supplementary Figure 5

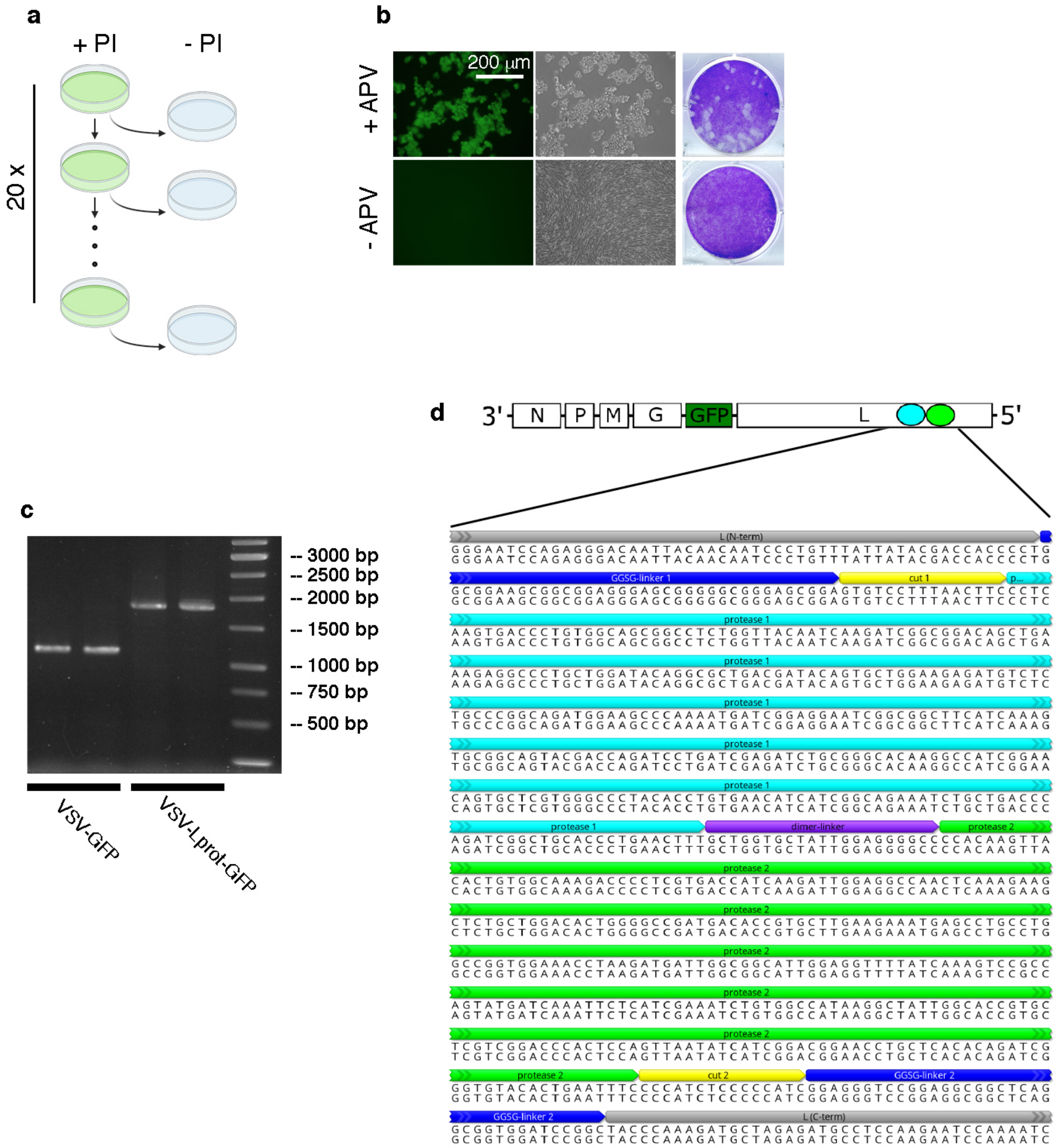

Supplementary Fig. 5. Genetic stability of protease inhibitor dependency of VSV-Lprot. VSV-Lprot-GFP was serially passaged 20 times on BHK-21 cells in the presence of either optimal (10 µM) or suboptimal (1 µM) doses of APV. In parallel, 5 µl of each supernatant was transferred onto dishes without APV. Experiment was performed with two independent passage lines **(a)**. After 20 passages GFP expression and plaque forming capacity remained dependent on presence of APV (**b**). Viral genomic RNA collected from passage P20 from the suboptimal amprenavir-treated virus propagation was purified, reverse transcribed and a PCR (primer pair L-for / L-rev) performed on the insert L-1620PR2 (size: 1831 bp). A VSV-GFP variant without protease insertion was used as control (size: 1114). Pairwise bands represent two independent passage studies **(c).** Sequencing of the insert after 20 passages revealed no additional mutation compared to starting sequence **(d)**. Schematic in **(a)** generated with BioRender. Source data are provided in the Source Data File.

Supplementary Figure 6

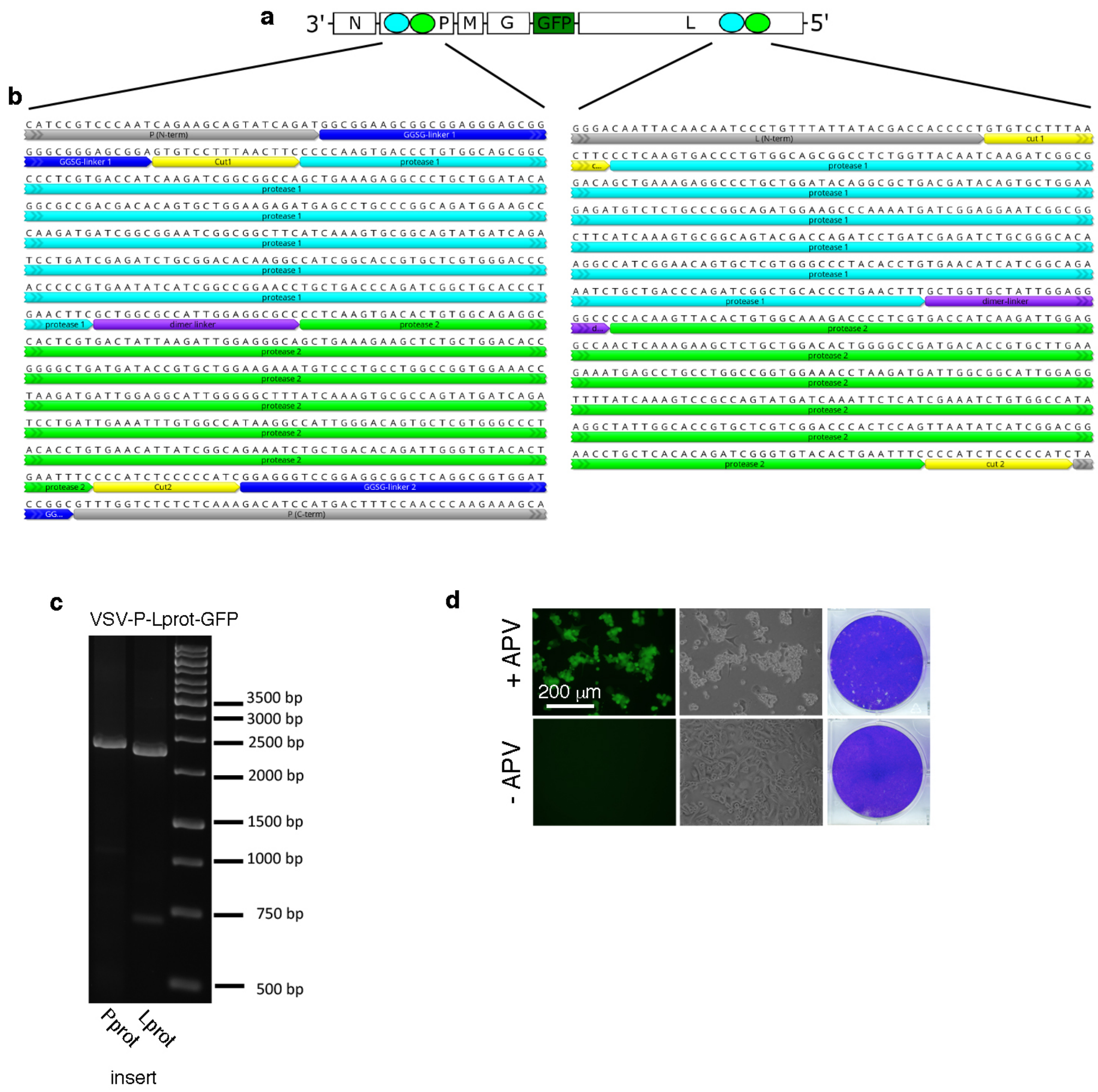

Supplementary Fig. 6. Dual insertion construct VSV-P-Lprot-GFP. Genome scheme of VSV-P-Lprot-GFP **(a)**. Two different codon usage variants of the protease dimer were used for insertions. Flexible GGSG linkers were included in the Pprot but not in the Lprot construct to minimize potential interactions. Purple: dimer linker; blue: GGSG linkers; yellow: HIV protease cleavage sequence; green / turquois: proteases; grey: insert-flanking viral genes P or L **(b)**. RNA purification, cDNA synthesis and PCR on insert adjacent regions of P (P-196PR2: 1829 bp) and L (L-1620PR2: 1759 bp) confirms successful integration of the corresponding protease dimer constructs **(c)**. VSV-P-Lprot-GFP infection and replication leads to cytopathic effects and GFP expression in the presence of protease inhibitors. Reduced plaque sizes reflecting the attenuated nature the double-prot variant **(d)**. Source data are provided in the Source Data File.

Supplementary Figure 7

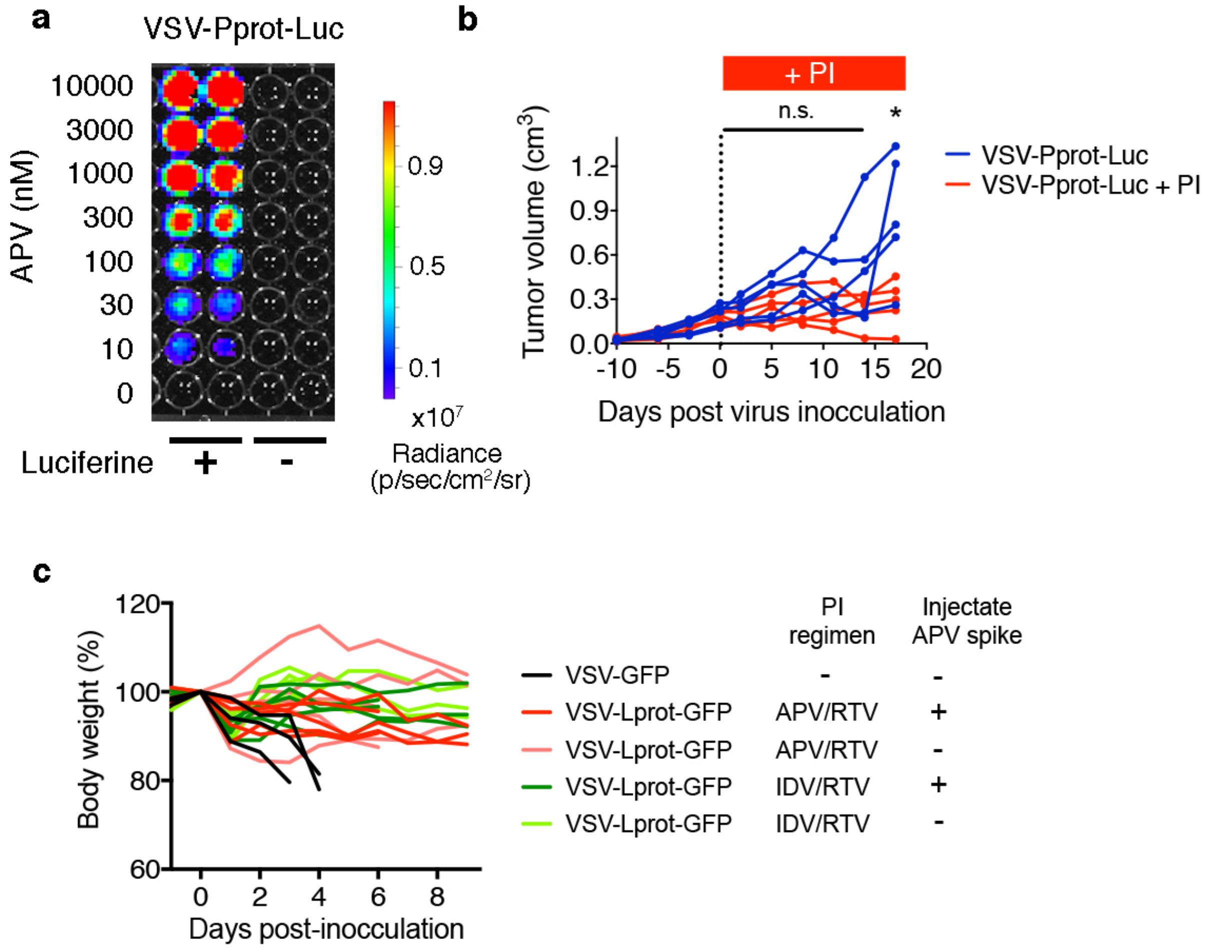

Supplementary Fig. 7. In vitro and in vivo activity of luciferase-expressing VSV-Pprot-Luc is dependent on protease inhibitor. a) BHK-21 cells were cultured in a 96-well plate and infected with VSV-Pprot-Luc at an MOI 1 and treated with increasing concentrations of APV. This study was performed two times. **b)** Subcutaneous human glioma U87 xenografts were intratumorally treated with luciferase-expressing VSV-Pprot-Luc with or without concomitant application of APV. Tumor growth curve from VSV-Pprot-Luc treated tumors in mice receiving PI (APV + RTV) (red) or drug vehicle (blue) (n=5; * = $p < 0.05$; unpaired two-tailed t test at indicated time points). **c)** Addition of 10 μM APV directly into the stereotactic injectate does not affect body weight in mice treated peripherally with a PI regimen containing APV/RTV or IDV/RTV, respectively. Source data are provided in the Source Data File.

Supplementary Figure 8

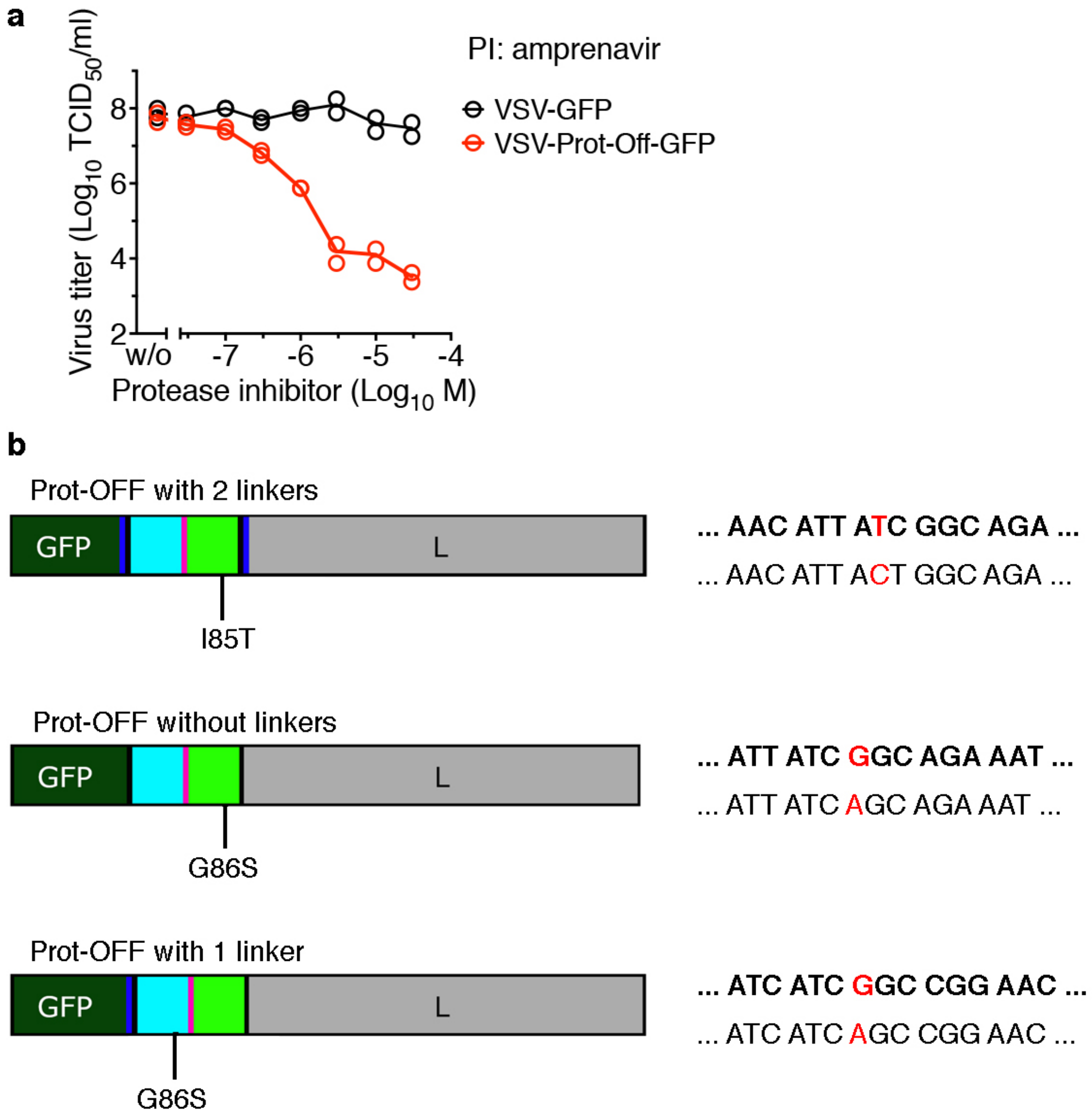

Supplementary Fig. 8. Amprenavir response and secondary mutations in VSV-Prot-OFF viruses. a) BHK cells were infected with VSV-GFP or VSV-Prot-OFF-GFP at an MOI of 1 in the presence of increasing doses of APV. Supernatant was collected 24 hrs later and virus progeny titer was determined via $TCID_{50}$ assay (data are mean ± SD; n = 2). **b)** Non-synonymous mutations in the HIV protease region of VSV-Prot-OFF variants with 2 linkers, 1 linker, or no linker between the protease linked dimer, GFP and polymerase (L). Amino acid change designation relates to numbering from first codon of respective protease. Reference nucleotide sequence in bold. Mutations in nucleotide sequence are highlighted in red. Source data are provided in the Source Data File.

Supplementary Figure 9

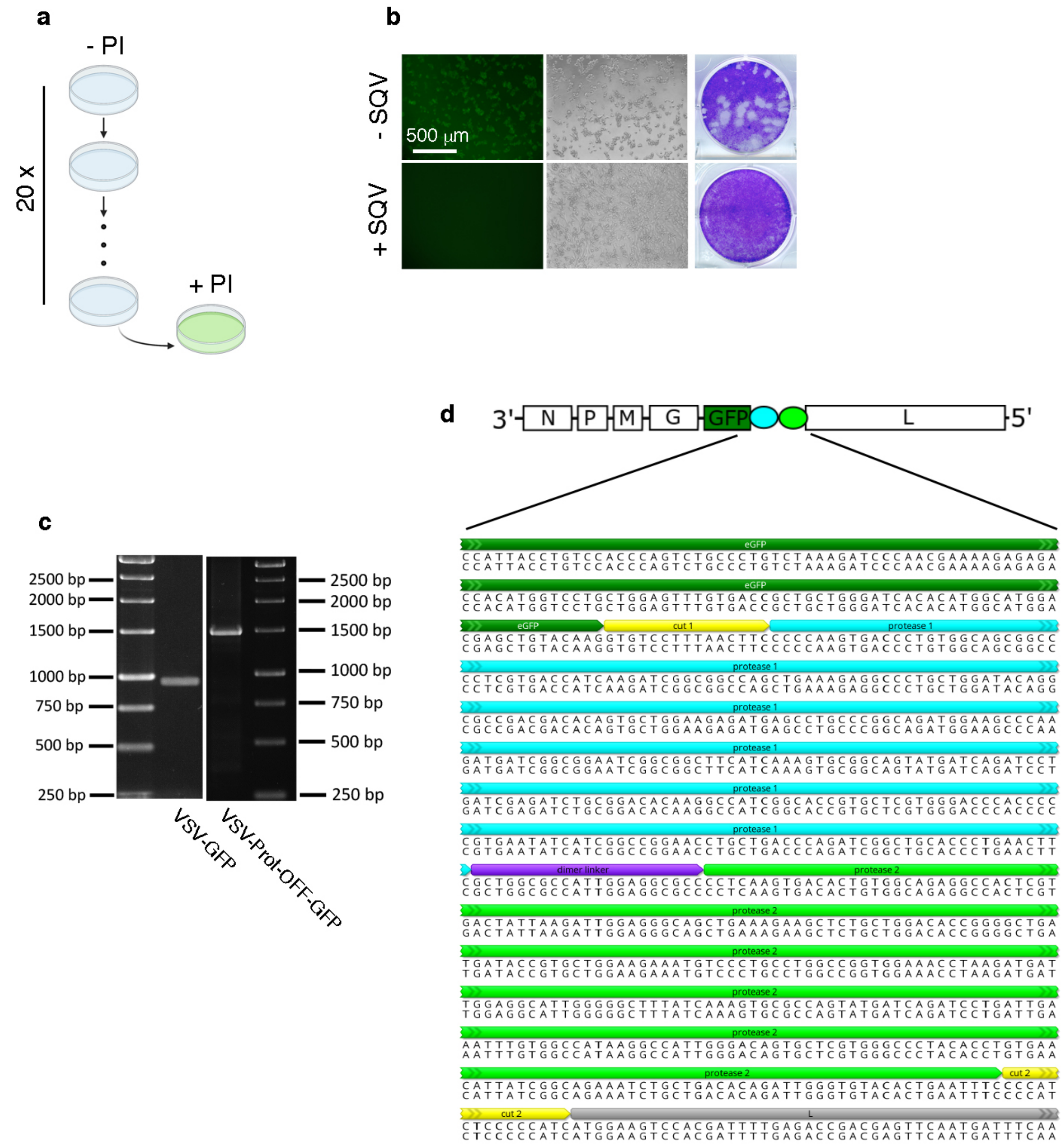

Supplementary Fig. 9. Genetic stability of protease inhibitor control of VSV-Prot-OFF-GFP. VSV-Prot-OFF-GFP was serially passaged 20 times on BHK-21 cells. Experiment was performed with two independent passage lines **(a)**. After 20 passages GFP expression and plaque forming capacity remained sensitive to blocking by treatment with protease inhibitor saquinavir (SQV) (**b**). Viral genomic RNA collected from passage P20 was purified, reverse transcribed and a PCR (primer pair GFP-33nt-before-MscI-for / L-N-term-rev) performed on the insert GFP-Prot-L (size: 1487 bp). A standard VSV-GFP virus with GFP positioned in the 5th position was used as control with the PCR fragment covering GFP, the intergeneic region (IGR), and L-protein (size 824 bp)**(c).** Sequencing of the insert after 20 passages revealed complete alignment with the starting sequence **(d)**. Schematic in **(a)** generated with BioRender. Source data are provided in the Source Data File.

Supplementary Table 1. Primer sequences and target regions.

name	sequence	target region
	**Prot-On** cloning	
N-35nt-before-BstZ17I-for	GCAAGGAATGCCCGACAGCC	VSV nucleoprotein
P-35nt-after-XbaI-rev	CGTCCGTCACCTCCGACAGAG	VSV phosphoprotein
GGSG-prot-for	GGCGGAAGCGGCGGAGGG	HIV protease linked dimer
GGSG-prot-rev	GCCGGATCCACCGCCTGAG	HIV protease linked dimer
L-1620-GGSG-rev	GCTCCCTCCGCCGCTTCCGCCAGGGGTGGTCGTATAATAAAC	VSV large protein
L-1620-GGSG-for	GGCTCAGGCGGTGGATCCGGCTACCCAAAGATGCTAGAGATG	VSV large protein
L-46nt-before-FseI-for	GCTGCCAAGTAATACACCGG	VSV large protein
L-48nt-after-SfoI-rev	TTTATCTCCTCCTAAAGTTTC	VSV large protein
L-1620-prot-rev	GGTCACTTGGGGGAAGTTAAAGGACACAGGGGTGGTCGTATAATAAAC	VSV large protein
L-1620-prot-for	CACTGAATTTCCCCATCTCCCCCATCTACCCAAAGATGCTAGAGATG	VSV large protein
prot-for	GTGTCCTTTAACTTCCCCCAAG	HIV protease linked dimer
prot-rev	GATGGGGGAGATGGGGAAATTC	HIV protease linked dimer
	**Prot-On** quality control	
P-for	GAGAGCAGAAATCCCAGTGGC	VSV phosphoprotein
M-rev	TAGAAGGGACGTTTCCCTGCCATT	VSV matrix protein
L-for	CATGCCGAGGACAGTTCTCTAT	VSV large protein
L-rev	ATTTCCTCCGACTCAAAGCAG	VSV large protein
	**Prot-Off** cloning and quality control	
GFP-33nt-before-MscI-for	ATCTGCACCACTGGAAAGCTC	Green fluorescent protein
GFP-GGSG-rev	CTCCCTCCGCCGCTTCCGCCCTTGTACAGCTCGTCCATGCC	Green fluorescent protein
$(GGSG)_3$-prot-for	GGCGGAAGCGGCGGAGGGAGCGGGGGCGGGAGCGGA GTGTCCTTTAACTTCCCCCAAG	HIV protease linked dimer
$(GGSG)_3$-prot-rev	GCCGGATCCACCGCCTGAGCCGCCTCCGGACCCTCC GATGGGGGAGATGGGGAAATTC	HIV protease linked dimer
GGSG-L-for	GCTCAGGCGGTGGATCCGGCATGGAAGTCCACGATTTTGAG	VSV large protein
GFP-cut1-rev	CTTGGGGGAAGTTAAAGGACACCTTGTACAGCTCGTCCATGCC	Green fluorescent protein
cut2-L-for	GAATTTCCCCATCTCCCCCATCATGGAAGTCCACGATTTTGAG	VSV large protein
L-N-term-rev	GATGTTGGGATGGGATTGGC	VSV large protein
	**VSV-luciferase** cloning	
XhoI-luc-for	GCAGTTCAGACTCGAGCCACCATGGAAGATGCC	photinus pyralis luciferase
NheI-luc-rev	GACGTCAGTTCGAGCTAGCTTATTACACGGCGATCTTGC	photinus pyralis luciferase
	**VSV-dsRed-ΔP** cloning	
XhoI-N-for	CGATCTCGAGGTATACATCTCTTACTACAGCAGG	VSV nucleoprotein
EcoRI-N-rev	CAGTGAATTCGATATCTGTTAGTTTTTTTCATATGTAGC	VSV nucleoprotein intergenic region
NotI-M-for	CGATGCGGCCGCACTATGAAAAAAAGTAACAGATATCACG	VSV matrix protein 3‘ intergenic region
SacII-M-rev	CAGTCCGCGGACGCGTAAACAGATCGATCTCTG	VSV matrix protein 5‘ intergenic region
