# Supplementary material for: Chemogenetic ON and OFF switches for RNA virus replication
Source: Nat Commun. 2021 Mar 1;12:1362. doi: 10.1038/s41467-021-21630-5 (PMC7921684; doi:10.1038/s41467-021-21630-5)
Supplement: Supplementary file 3 — Reporting Summary [file 41467_2021_21630_MOESM3_ESM.pdf]

nature research

Corresponding author(s): Guido Wollmann, Dorotheé von Laer

Last updated by author(s): Jan 18, 2021

# Reporting Summary

Nature Research wishes to improve the reproducibility of the work that we publish. This form provides structure for consistency and transparency in reporting. For further information on Nature Research policies, see Authors & Referees and the Editorial Policy Checklist .

## Statistics

For all statistical analyses, confirm that the following items are present in the figure legend, table legend, main text, or Methods section.

| n/a | Confirmed | |
|---|---|---|
| ☐ | ☒ | The exact sample size (*n*) for each experimental group/condition, given as a discrete number and unit of measurement |
| ☐ | ☒ | A statement on whether measurements were taken from distinct samples or whether the same sample was measured repeatedly |
| ☐ | ☒ | The statistical test(s) used AND whether they are one- or two-sided<br>*Only common tests should be described solely by name; describe more complex techniques in the Methods section.* |
| ☒ | ☐ | A description of all covariates tested |
| ☐ | ☒ | A description of any assumptions or corrections, such as tests of normality and adjustment for multiple comparisons |
| ☐ | ☒ | A full description of the statistical parameters including central tendency (e.g. means) or other basic estimates (e.g. regression coefficient) AND variation (e.g. standard deviation) or associated estimates of uncertainty (e.g. confidence intervals) |
| ☐ | ☒ | For null hypothesis testing, the test statistic (e.g. *F*, *t*, *r*) with confidence intervals, effect sizes, degrees of freedom and *P* value noted<br>*Give P values as exact values whenever suitable.* |
| ☒ | ☐ | For Bayesian analysis, information on the choice of priors and Markov chain Monte Carlo settings |
| ☒ | ☐ | For hierarchical and complex designs, identification of the appropriate level for tests and full reporting of outcomes |
| ☒ | ☐ | Estimates of effect sizes (e.g. Cohen's *d*, Pearson's *r*), indicating how they were calculated |

*Our web collection on statistics for biologists contains articles on many of the points above.*

## Software and code

Policy information about availability of computer code

| | |
|---|---|
| Data collection | Modelling: I-TASSER 5.1<br>Luciferase imaging: Caliper Live Sciences-Living Image® Software 4.3.1.<br>Microscopy: NIS-Elements BR 4.20.01, VisiView software (Visitron) 4.1.0.3. |
| Data analysis | Statistics: GraphPad Prism 8<br>Sequence analysis: Geneious Prime 2019.2.1<br>Structure analysis: Chimera 1.12, Coot 0.8.7.1<br>Luciferase quantification: Caliper Live Sciences-Living Image® Software 4.3.1.<br>Figure generation and processing: Adobe Photoshop CS6, Inkscape 0.92.3 |

For manuscripts utilizing custom algorithms or software that are central to the research but not yet described in published literature, software must be made available to editors/reviewers. We strongly encourage code deposition in a community repository (e.g. GitHub). See the Nature Research guidelines for submitting code & software for further information.

## Data

Policy information about availability of data

All manuscripts must include a data availability statement. This statement should provide the following information, where applicable:

- Accession codes, unique identifiers, or web links for publicly available datasets
- A list of figures that have associated raw data
- A description of any restrictions on data availability

All pertinent data to support this study are included in the manuscript and supplementary files. Further data supporting the findings are available upon request. The sequences of the prot constructs have been deposited at NCBI's GenBank with the accession codes MW316665 (not codon optimized), MW316666 (codon optimized).

# Field-specific reporting

Please select the one below that is the best fit for your research. If you are not sure, read the appropriate sections before making your selection.

☒ Life sciences ☐ Behavioural & social sciences ☐ Ecological, evolutionary & environmental sciences

For a reference copy of the document with all sections, see nature.com/documents/nr-reporting-summary-flat.pdf

# Life sciences study design

All studies must disclose on these points even when the disclosure is negative.

| | |
|---|---|
| Sample size | Sample sizes were chosen empirically based on experience from our previous studies using the presented tumor models with (PMID: 24812275) or without (PMID: 29635363) oncolytic virotherapies, which were found to meet estimated statistical power. The "3R's" were adhered to. Reporting according to ARRIVE guidlines was applied. |
| Data exclusions | No experimental data were excluded after initiation of treatments. In one of our stereotactic brain injection studies (presented in Fig 2i and S7c), one VSV-GFP control animal had to be sacrificed during the procedure due to complications from surgery. This was unrelated to the treatment and the mouse was excluded from the study. |
| Replication | In-vitro experiments were replicated at least once (n equal or greater than 2) as indicated in the figure legends. In-vivo experiments were not replicated with the same design, although key treatment and control groups for the intracranial injection study to assess neurotoxicity were repeated across two independent experiments. |
| Randomization | Mice were allocated into different treatment groups one day before treatment initiation. By evenly distributing larger and smaller tumors, a comparable median tumor size was in place at the start of the intervention. |
| Blinding | For most parts, investigators were not blinded during data collection or analysis because planning, execution, and analysis of the studies was performed by the same personnel. Histological analysis of intracranial (Fig 2h,J) and intratumoral spread (Fig 3 h, i, j) was performed blinded via coded specimen numbers. |

# Reporting for specific materials, systems and methods

We require information from authors about some types of materials, experimental systems and methods used in many studies. Here, indicate whether each material, system or method listed is relevant to your study. If you are not sure if a list item applies to your research, read the appropriate section before selecting a response.

## Materials & experimental systems

| n/a | Involved in the study | |
|---|---|---|
| ☐ | ☒ | Antibodies |
| ☐ | ☒ | Eukaryotic cell lines |
| ☒ | ☐ | Palaeontology |
| ☐ | ☒ | Animals and other organisms |
| ☒ | ☐ | Human research participants |
| ☒ | ☐ | Clinical data |

## Methods

| n/a | Involved in the study | |
|---|---|---|
| ☒ | ☐ | ChIP-seq |
| ☒ | ☐ | Flow cytometry |
| ☒ | ☐ | MRI-based neuroimaging |

## Antibodies

| | |
|---|---|
| Antibodies used | Primary antibody α-VSV-N (10G4, #EB009, raised in mouse, Kerafast, Inc., Boston, USA)<br>Secondary antibody goat-α-mouse IgG2a Alexa 594 (A-21135, ThermoFisher, Vienna, Austria) |
| Validation | The primary antibody was initially described by the laboratory of Douglas S. Lyles, PhD, Wake Forest School of Medicine. Primary antibody used in this study was manufactured by Kerafast using Absolute Antibody's Recombinant Platform with variable regions (i.e., specificity) from the hybridoma 10G4.<br><br>Relevant citations:<br>Lyles DS, Puddington L, McCreedy BJ Jr. Vesicular stomatitis virus M protein in the nuclei of infected cells. J Virol. 1988 Nov;62 (11):4387-92. PubMed PMID: 2845149<br><br>Lefrancios L, Lyles DS. The interaction of antibody with the major surface glycoprotein of vesicular stomatitis virus. I. Analysis of neutralizing epitopes with monoclonal antibodies. Virology 121: 157-167, 1982.<br><br>Malikov V, da Silva ES, Jovasevic V, Bennett G, de Souza Aranha Vieira DA, Schulte B, Diaz-Griffero F, Walsh D, Naghavi MH. HIV-1 capsids bind and exploit the kinesin-1 adaptor FEZ1 for inward movement to the nucleus. Nat Commun. 2015 Mar 30;6:6660. |

doi: 10.1038/ncomms7660. PubMed PMID: 25818806; PubMed Central PMCID: PMC4380233.

Bresk CA, Hofer T, Wilmschen S, Krismer M, Beierfuß A, Effantin G, Weissenhorn W, Hogan MJ, Jordan APO, Gelman RS, Montefiori DC, Liao HX, Schmitz JE, Haynes BF, von Laer D, Kimpel J. Induction of Tier 1 HIV Neutralizing Antibodies by Envelope Trimers Incorporated into a Replication Competent Vesicular Stomatitis Virus Vector. Viruses. 2019 Feb 15;11(2). pii: E159.

Hulswit RJG, Lang Y, Bakkers MJG, et al. Human coronaviruses OC43 and HKU1 bind to 9-O-acetylated sialic acids via a conserved receptor-binding site in spike protein domain A. Proc Natl Acad Sci U S A. 2019;116(7):2681–2690. View ArticleTortorici MA, Walls AC, Lang Y, Wang C, Li Z, Koerhuis D, Boons GJ, Bosch BJ, Rey FA, de Groot RJ, Veesler D. Structural basis for human coronavirus attachment to sialic acid receptors. Nat Struct Mol Biol. 2019 Jun;26(6):481-489.

## Eukaryotic cell lines

Policy information about cell lines

| | |
|---|---|
| Cell line source(s) | BHK-21 cells (American Type Culture Collection (ATCC), Manassas, VA),<br>293T cells (ATCC) were used to generate 293tsA1609neo and 293-VSV (293 expressing N, P-GFP and L of VSV - PMID: 20181705)<br>G62 cells were a gift from M. Westphal (University Hospital Eppendorf, Hamburg, Germany) and are referenced in PMID: 8154317<br>U87 cells (ATCC) |
| Authentication | All cell lines were checked by morphology compared to cell bank references. Genomic authentication was not performed. |
| Mycoplasma contamination | All cell lines were tested negative for mycoplasma contamination |
| Commonly misidentified lines (See ICLAC register) | No commonly misidentified cell lines were used in this study. |

## Animals and other organisms

Policy information about studies involving animals; ARRIVE guidelines recommended for reporting animal research

| | |
|---|---|
| Laboratory animals | Six- to eight-week old female NOD.CB-17-Prkdcscid/Rj and athymic nude mice were purchased from Janvier Labs (Le Genest-Saint-Isle, France) and housed in a BL2 facility with a 12-hour light/dark cycle with unrestricted access to food and water. Temperature in animal facilities was 20-24 °C for NOD.CB-17-Prkdcscid/Rj and 22-26 °C for athymic nude mice, humidity was 55 ± 10%. |
| Wild animals | No wild animals were used in this study. |
| Field-collected samples | No field-collected samples were used in this study. |
| Ethics oversight | Animal experiments were approved by the institutional animal care and use review board and the Austrian Ministry of Science. |

Note that full information on the approval of the study protocol must also be provided in the manuscript.
